# Exceptional longevity and muscle and fitness related genotypes: a functional *in vitro* analysis and case-control association replication study with SNPs *THRH* rs7832552, *IL6* rs1800795, and *ACSL1* rs6552828

**DOI:** 10.3389/fnagi.2015.00059

**Published:** 2015-05-06

**Authors:** Noriyuki Fuku, Zi-hong He, Fabian Sanchis-Gomar, Helios Pareja-Galeano, Ye Tian, Yasumichi Arai, Yukiko Abe, Haruka Murakami, Motohiko Miyachi, Hirofumi Zempo, Hisashi Naito, Thomas Yvert, Zoraida Verde, Letizia Venturini, Carmen Fiuza-Luces, Alejandro Santos-Lozano, Gabriel Rodriguez-Romo, Giovanni Ricevuti, Nobuyoshi Hirose, Enzo Emanuele, Nuria Garatachea, Alejandro Lucia

**Affiliations:** ^1^Graduate School of Health and Sports Science, Juntendo UniversityChiba, Japan; ^2^Biology Centre, China Institute of Sport ScienceBeijing, China; ^3^Research Institute of Hospital 12 de Octubre (“i+12”)Madrid, Spain; ^4^School of Doctorate Studies and Research, European University of MadridMadrid, Spain; ^5^Center for Supercentenarian Study, Keio University School of MedicineTokyo, Japan; ^6^Department of Health Promotion and Exercise, National Institute of Health and NutritionTokyo, Japan; ^7^Department of Health Sciences, University of PaviaPavia, Italy; ^8^INEF, Universidad PolitécnicaMadrid, Spain; ^9^Department of Physiotherapy and Nursing, Faculty of Health and Sport Science, University of ZaragozaHuesca, Spain

**Keywords:** centenarians, polymorphisms, luciferase reporter, gene association study, muscle and sarcopenia

## Abstract

There are several gene variants that are candidates to influence functional capacity in long-lived individuals. As such, their potential association with exceptional longevity (EL, i.e., reaching 100+ years) deserves analysis. Among them are rs7832552 in the thyrotropin-releasing hormone receptor (*TRHR*) gene, rs1800795 in the interleukin-6 (*IL6*) gene and rs6552828 in the coenzyme A synthetase long-chain 1 (*ACSL1*) gene. To gain insight into their functionality (which is yet unknown), here we determined for the first time luciferase gene reporter activity at the muscle tissue level in rs7832552 and rs6552828. We then compared allele/genotype frequencies of the 3 abovementioned variants among centenarians [*n* = 138, age range 100–111 years (114 women)] and healthy controls [*n* = 334, 20–50 years (141 women)] of the same ethnic and geographic origin (Spain). We also studied healthy centenarians [*n* = 79, 100–104 years (40 women)] and controls [*n* = 316, 27–81 years (156 women)] from Italy, and centenarians [*n* = 742, 100–116 years (623 women)] and healthy controls [*n* = 499, 23–59 years (356 women)] from Japan. The *THRH* rs7832552 *T*-allele and *ACSL1* rs6552828 *A*-allele up-regulated luciferase activity compared to the *C* and *G*-allele, respectively (*P* = 0.001). Yet we found no significant association of EL with rs7832552, rs1800795 or rs6552828 in any of the 3 cohorts. Further research is needed with larger cohorts of centenarians of different origin as well as with younger old people.

## Introduction

The oldest old population (≥85 years) is rapidly expanding among westerners (Waite, 2004; Robine and Paccaud, 2005). However, aging is associated with an increased risk of loss of functional independence (Christensen et al., 2008). In this regard, centenarians (people aged 100+ years) are the paradigm of healthy aging, as they have usually postponed (or even avoided, in some cases) major age-related diseases, as well as the onset of disability, until they were well into their nineties (Terry et al., 2008). Thus, the search for the gene variants that might influence the likelihood of reaching exceptional longevity (EL, i.e., becoming a centenarian) might help identify targets of “anti-aging” interventions.

In old people, functional independence is dependent on physical fitness, which in turn is determined by several phenotypes such as mainly cardiorespiratory fitness and muscle performance (Garber et al., 2011). Concerning the latter, aging is inevitably associated with a decline in muscle mass and function, i.e., sarcopenia, with an acceleration of this process increasing the risk of mortality (Metter et al., 2004; Ruiz et al., 2008). Thus, those gene variations that can be associated with preservation of muscle mass/function at advanced ages, e.g., the K153R polymorphism in the myostatin (*MSTN*) gene (Garatachea et al., 2013a) or the I/D polymorphism in the angiotensin converting enzyme (*ACE*) (Garatachea et al., 2013b) as well as other genetic variations linked with muscle aerobic capacity (e.g., the R577X mutation in the α-actinin-3 (*ACTN3*) gene), might also influence the likelihood of reaching EL (Fiuza-Luces et al., 2011).

There are other potential candidates to influence muscle phenotypes in long-lived individuals and as such their potential association with EL deserves analysis. In this regard, a recent genome wide scan (GWAS) analysis of 379,319 SNPs in US Caucasians of both genders (aged 50 years on average) revealed an association of lean body mass with 2 SNPs in tight linkage disequilibrium within the thyrotropin-releasing hormone receptor (*TRHR*) gene, rs16892496 and rs7832552 (Liu et al., 2009). These results were further corroborated in independent cohorts of older Caucasians of both genders, aged 63 (men) and 61 years (women). The functional significance of these 2 variants remains however to be determined. Another candidate is the −174C/*G* polymorphism (rs1800795) in the interleukin-6 (*IL6*) gene, where the *G*-allele is associated with higher transcription *in vitro* (Fishman et al., 1998) and *in vivo* conditions (Bennermo et al., 2004). IL6 is a multifunctional cytokine that might be also involved in muscle regeneration (Serrano et al., 2008). Pereira et al recently found that the *GG* genotype, which is associated with lower IL6 levels and thus with “anti-inflammatory” profile, was associated with better physical performance in community-dweller elderly women (≥65 years) (Pereira et al., 2013).

As for cardiorespiratory fitness [which is usually determined with peak oxygen uptake (VO_2peak_)], a recent GWAS study in sedentary Caucasians found that, among 324,611 SNPs, the strongest association with the VO_2peak_ response to exercise was found to acyl coenzyme A synthetase long-chain 1 (*ACSL1*) gene polymorphism rs6552828 (Bouchard et al., 2011). The *ACSL1* gene is a candidate to explain individual variability in VO_2peak_, as well as in some health-related phenotypes, owing to its potential role in aerobic metabolism at the adypocyte, cardiomyocyte, liver and skeletal muscle fiber level (Martin et al., 1997; Coleman et al., 2000; Hall et al., 2003; Mashek et al., 2006; Ellis et al., 2010), yet its functional significance has not been assessed.

In order to analyze their functionality at the muscle level, we measured for the first time luciferase gene reporter activity in *TRHR* rs16892496 and rs7832552, and also in *ACSL1* rs6552828. Only rs7832552 in the *TRHR* gene was genotyped in this study because it is in tight linkage disequilibrium with rs16892496 (Liu et al., 2009). Based on the hypothesis that common genetic polymorphisms influencing physical fitness may also have an impact on the ability to reach EL, we then compared allele/genotype frequencies of the abovementioned SNPs—together with the *IL6* rs1800795 SNP (whose functional significance is already known, as mentioned above)—among Spanish centenarians (*cases*) and healthy controls matched by ethnic and geographic origin and also in 2 other geographically and ethnically-independent replication cohorts (from Italy and Japan).

## Methods

### Functional analysis: luciferase reporter gene

The fragment, including the allele, was directly inserted into the pGL3-promoter at the restriction recognition sites *MluI/NheI* in the 5′ and *XhoI* in the 3′ (see below -in bold) of the sequences obtained from the genomes of:

- one individual homozygous for the rs16892496 *A*-allele and one individual homozygous for the rs16892496 *C*-allele (see below -underlined)
  rs16892496 *AA*
  GCTAGCTATGAAAGATCTACGTTAAAACATAAGGTTAAGCTGTGCAGTGTACAGAAGAGACAAGAAAGTGGTACTTACTGTGCATAAGGTTGAAGAGCAAGCCCCC**A**GTGGGATACAAGTCACTCTCAGGCTTGAAAAATGAGTAGGCATTCACTAGGCCAACATAAAATACAAGAAGACCCTCCAGTCTGCAGAAGTAGTCAATGACTCGAG
  rs16892496 *CC*
  GCTAGCTATGAAAGATCTACGTTAAAACATAAGGTTAAGCTGTGCAGTGTACAGAAGAGACAAGAAAGTGGTACTTACTGTGCATAAGGTTGAAGAGCAAGCCCCC**C**GTGGGATACAAGTCACTCTCAGGCTTGAAAAATGAGTAGGCATTCACTAGGCCAACATAAAATACAAGAAGACCCTCCAGTCTGCAGAAGTAGTCAATGACTCGAG
- one individual homozygous for the rs7832552 *C*-allele and one homozygous for the rs7832552 *T*-allele (see below -underlined)
  rs7832552-*CC*
  GAGCTCATTAGCCTTGTGACAAAAGCAACGCACTCCATTTTGCACACAGTACTTGACTTTATTTTGCTACTGCCTTGACCTCAAAGGAATGTGATAGTGTGAGGTA**C**GAATGCTCTTAATAAACAGGATCGATCAAGGGTGCTTGACTCTTGTTGTTCATGTGCAAGTATAGTGGCTTTTTTGTGCCTCAACAAAACCATCAAGAGTCTCGAG
  rs7832552-*TT*
  GAGCTCATTAGCCTTGTGACAAAAGCAACGCACTCCATTTTGCACACAGTACTTGACTTTATTTTGCTACTGCCTTGACCTCAAAGGAATGTGATAGTGTGAGGTATGAATGCTCTTAATAAACAGGATCGATCAAGGGTGCTTGACTCTTGTTGTTCATGTGCAAGTATAGTGGCTTTTTTGTGCCTCAACAAAACCATCAAGAGTCTCGAG
- one individual homozygous for the rs6552828 *A*-allele and one individual homozygous for the rs6552828 *G*-allele (see below -underlined)
  rs6552828-AA
  GAGCTCCAAGACATTATAGCCAAAAGAAACAAACAGATAAATTGGTGTGCATAAACTTTAAACCAACCACCAGATATCTAAAGAGGGAATACAGCACAGTGTTGGA**A**AGAAAGTACAGAATAGTATTTGAGATCCTAGATGCAGCCGGACGCGGTGGCTCATGCCTGTAATCCCAGCACTTTGGGAAGCCGAGGCGGGTGGATCACCCTCGAG
  rs6552828-GG
  GAGCTCCAAGACATTATAGCCAAAAGAAACAAACAGATAAATTGGTGTGCATAAACTTTAAACCAACCACCAGATATCTAAAGAGGGAATACAGCACAGTGTTGGA**G**AGAAAGTACAGAATAGTATTTGAGATCCTAGATGCAGCCGGACGCGGTGGCTCATGCCTGTAATCCCAGCACTTTGGGAAGCCGAGGCGGGTGGATCACCCTCGAG

We used mice skeletal muscle C2C12 cell lines to study muscle-specific expression. We performed cell cultures, transfections and dual-luciferase reporter assays following the procedures previously reported by our group (He et al., 2011). We used the pRL-SV40 vector as an internal control for variations in transfection efficiency, and the pGL3-promoter vector without an insert as a negative control. The transfected cells were harvested after 48 h, and assayed for firefly and renilla luciferase activity with the dual-luciferase reporter assay system (Promega Biotech, Beijing, China) using a luminometer following the manufacturer's recommendations (TecanGenios Pro, Männedorf, Switzerland). From each measurement, we divided firefly by renilla luciferase activity reading to calculate relative luciferase activity. We performed the experiments in triplicates and expressed relative luciferase activity values as the means±SD of the 3 different measurements.

### Subjects

We obtained approval from the local ethics committees [European University of Madrid (Spain), University of Pavia (Italy), and National Institute of Health and Nutrition, (Japan) Medical Research Institute and Keio University (Japan)] and the study followed the tenets of the Declaration of Helsinki for Human Research. Written consent was also obtained from each participant.

#### Spanish cohort

Two groups of Spanish subjects were assessed: (i) 138 cases (centenarians, aged 100–111 years, 114 women); and (ii) 334 healthy controls (aged 20–50 years, 141 women). All the subjects were of the same Caucasian (Spanish) descent for ≥3 generations. The major diseases among the centenarians were osteoarthritis (66%), hypertension (57%), dementia (51%) and cardiovascular disease (CVD, 29%). The DNA of a convenience sample of 355 younger disease-free controls with no reported family history of high longevity (>90 years) was collected during 2008-2012 in the European University of Madrid.

#### Italian cohort

Two groups of subjects from Northern Italy (mainly from Lombardy and Piedmont) were studied: (i) 79 cases (healthy centenarians, aged 100–104 years, 40 women); and (ii) 316 healthy controls (aged 27–81 years, 156 women). All patients and controls were Caucasian whites of Italian descent for ≥3 generations The Italian centenarians were free of major age-related diseases, i.e., severe cognitive impairment, clinically evident cancer, CVD, renal insufficiency or severe physical impairment (Emanuele et al., 2010). Controls were free of CVD or cerebrovascular disease, cancer, dementia, chronic autoimmune/ inflammatory disorders, renal or hepatic failure, and major psychiatric conditions.

#### Japanese cohort

Two groups of subjects of the same Asian (Japanese) descent were assessed: (i) 742 cases (centenarians, aged 100–116 years, 623 women); and (ii) 499 healthy controls (aged 23–59 years, 356 women). The group of cases was gathered from 2 cohorts, which are described in detail elsewhere (Gondo et al., 2006): the Tokyo Centenarians Study (TCS) and the Semi-Supercentenarians Study in Japan (SSC-J). The prevalence of hypertension, CVD and dementia among the Japanese centenarians was of 63.6, 28.8, and 59.4%, respectively. Controls from both genders (aged <60 years, and free of diagnosed CVD and chronic renal failure) were recruited during years 2008–2012.

### Genotyping

As mentioned above, only one *THRH* SNP, rs7832552, was genotyped in the 3 cohorts (and not rs16892496) because the genotype distributions of both SNPs are completely linked according to available HapMap for both European and Asian populations (sorted as a Supplementary file 1) and previous research has shown that both SNPs are in strong linkage disequilibrium (*r*^2^ = 0.98). All genotyping was performed only for research purposes with the researchers who performed the genotyping being blinded to the participants' identities. For quality control, a random ~20% of the samples of each cohort were genotyped again, with no differences in the results compared with the initial genotyping.

#### Spanish cohort

DNA was extracted from the participants' buccal cells (saliva samples) using a standard phenol chloroform protocol and the genotype analyses were performed in the Biomedicine laboratory at the European University, Madrid (Spain). The DNA samples were diluted with sterile water and stored at −20°C until analysis. Genotyping was performed by Real-Time PCR and using the TaqMan® rs7832552, rs6552828, and rs1800795 SNP genotyping assays with a Step One Real-Time PCR System (Applied Biosystems, Foster City, CA).

#### Italian cohort

Genomic DNA was purified from blood leukocytes using the QiaAmp DNA Mini kit (Qiagen, Hilden, Germany) according to the manufacturer's protocol. Genotyping was performed at the Cellular Pathophysiology and Clinical Immunology Laboratory (University of Pavia) using the TaqMan® rs7832552, rs6552828, and rs1800795 SNP genotyping assays (Applied Biosystems, Foster City, CA, USA).

#### Japanese cohort

Total genomic DNA was extracted from blood leukocytes with a QIAamp DNA Blood Mini or Maxi Kit (Qiagen, Tokyo, Japan). Genotyping of rs7832552, rs6552828, and rs1800795 was performed at the Institute of Health and Sports Science and Medicine (Juntendo University) with Real Time Thermocycler (LightCycler 480, Roche Applied Science, Mannheim, Germany) using TaqMan SNP genotyping assay method. PCR 384-well plates were read on LightCycler 480 using the end-point analysis mode. Allelic discrimination analysis was performed with a LightCycler 480 SW software version 1.5.1.62 (Roche Applied Science, Mannheim, Germany).

### Statistical analysis

One-way analysis of variance was used to compare the relative luciferase activity in the different plasmids of each SNP. Allele frequencies were calculated by gene-counting. We tested Hardy-Weinberg equilibrium (HWE) using χ^2^-test. Genotype/allele frequencies of cases vs. controls within each cohort (Spanish, Italian, and Japanese) were compared using the χ^2^-test with Yates' correction and the association between genotypes/alleles and EL within each of the 3 cohorts was analyzed with logistic regression analysis after adjusting for sex. All statistical analyses were performed using the PASW (v. 18.0 for WINDOWS, Chicago) and corrected for multiple comparisons using the Bonferroni's method -that is, the threshold *P*-value was obtained by dividing 0.05 by the number of studied polymorphisms (*P* = 0.05/3 = 0.017).

## Results

### Functional analysis

**Figure 1:**
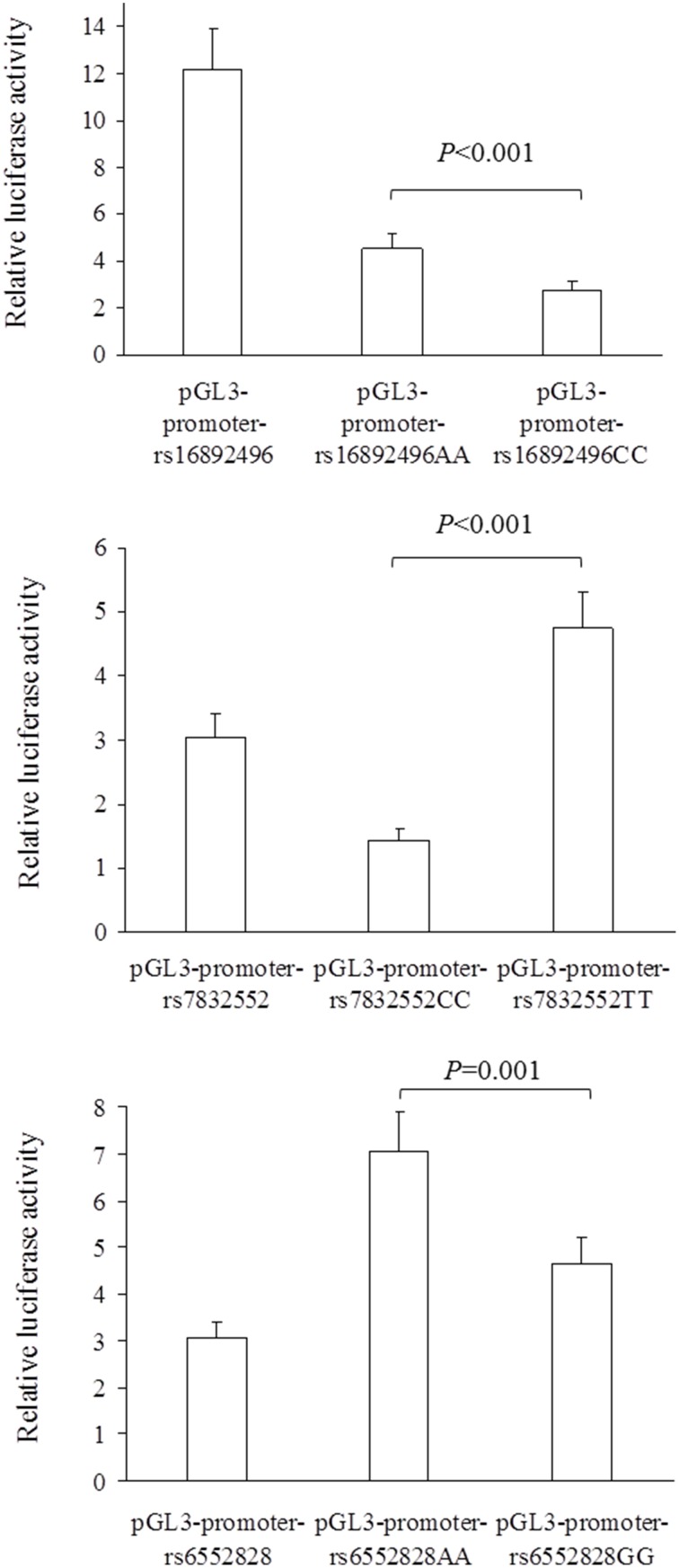
**Comparison of relative luciferase activity (i.e., firefly luciferase activity divided by renilla luciferase activity) between plasmids for the *THRH* rs16892496 (upper panel), *THRH* rs7832552 (middle panel), and *ACSL1* rs6552828 (lower panel)**. Values are mean ± SD of three different experiments, each performed in triplicate. For the 3 SNPs, statistical significance was reached for all the comparisons between plasmids (all *P* ≤ 0.001).

The results of luciferase report analyses are presented in Figure 1. All the SNPs we studied showed functional significance, as reflected by differences in luciferase activity between the 3 SNP constructs (all *P* ≤ 0.001); the *THRH* rs16892496 *A*-allele up-regulated luciferase activity compared to the *C*-allele (upper panel), the *THRH* rs7832552 *T*-allele up-regulated luciferase activity compared to the *C*-allele (middle panel), and the *ACSL1* rs6552828 *A*-allele up-regulated luciferase activity compared to the *G*-allele (lower panel).

### Spanish cohort

Rate of genotyping success was as follows: *THRH* rs7832552, 97.2% in cases and 100% in controls; *ACSL* rs6552828, 97.2% in cases and 99.1% in controls; *IL6* rs1800795, 100% in cases and 94.9% in controls. The distribution of all genotypes was consistent with the HWE in both groups (*P* > 0.05), except for *IL6* rs1800795 in the control group (*P* < 0.01).

The results of genotype/allele frequency distributions as well as of binary logistic regression adjusted by sex are shown in Table 1 and summarized below. The allele (χ^2^ = 1.21, *P* = 0.27) or genotype frequency distributions of *THRH* rs7832552 did not differ between cases and controls (χ^2^ = 2.74, *P* = 0.25). Using logistic regression analysis, no significant associations were found between EL and rs7832552, including when analyzing both sexes separately (data not shown). No differences were found for *IL6* rs1800795 in allele (χ^2^ = 1.01, *P* = 0.32) or genotype distributions (χ^2^ = 3.89, *P* = 0.14), with no significant association with EL after adjusting for sex or when analyzing both sexes separately -data not shown) or for *ACSL1* rs6552828 (χ^2^ = 2.28, *P* = 0.13 for allele distribution and χ^2^ = 3.30, *P* = 0.19 for genotype distribution, with no significant association with EL after adjusting for sex or when analyzing both sexes separately -data not shown).

**Table 1:** **Genotype/allele frequencies of *THRH* rs7832552, *ACSL1* rs6552828 and *IL6* rs1800795 and results of logistic regression analysis, in the Spanish cohort**.

		**Controls**		**Cases**		**OR**	**95%CI**	***P*-value**
		**N**	**%**	**N**	**%**			
**rs7832552**								
Codom	*CC*	174	52.1	61	44.2	1.00		0.14
	*CT*	135	40.4	67	48.5	1.52	0.97–2.37	
	*TT*	25	7.5	10	7.3	1.71	0.72–4.09	
Dom	*CC*	174	52.1	61	44.2	1.00		0.048
	*CT-TT*	160	47.9	77	55.8	1.54	1.00–2.37	
Reces	*CC-CT*	309	92.5	128	92.8	1.00		0.44
	*TT*	25	7.5	10	7.2	1.40	0.60–3.24	
Overdom	*CC-TT*	199	59.6	71	51.5	1.00		0.11
	*CT*	135	40.4	67	48.5	1.42	0.92–2.19	
Log-additive		–	–	–	–	1.41	0.99–1.99	0.055
Allele	*C*	483	72	189	68	1.00		0.06
	*T*	185	28	87	32	1.37	0.98–1.97	
**rs6552828**								
Codom	*GG*	126	38.1	65	47.1	1.00		0.41
	*GA*	153	46.2	54	39.1	1.36	0.86–2.16	
	*AA*	52	15.7	19	13.8	1.25	0.65–2.40	
Dom	*GG*	126	38.1	65	47.1	1.00		0.19
	*GA-AA*	205	61.9	73	52.9	1.33	0.87–2.05	
Reces	*GG-GA*	279	84.3	119	86.2	1.00		0.82
	*AA*	52	15.7	19	13.8	1.07	0.58–1.98	
Overdom	*GG-AA*	178	53.8	84	60.9	1.00	0.83–1.99	0.25
	*GA*	153	46.2	54	39.1	1.29	0.86–1.60	
Log-additive		–	–	–	–	1.18	0.86–1.60	0.30
Allele	*G*	405	61	184	67	1.00		0.29
	*A*	257	39	92	33	0.84	0.61–1.15	
**rs1800795**								
Codom	*GG*	170	53.6	65	45.5	1.00		0.20
	*GC*	101	31.9	59	41.3	0.65	0.41–1.04	
	*CC*	46	14.5	19	13.3	0.82	0.43–1.58	
Dom	*GG*	170	53.6	65	45.5	1.00		0.09
	*GC-CC*	147	46.4	78	54.5	0.70	0.45–1.07	
Reces	*GG-GC*	271	85.5	124	86.7	1.00		0.96
	*CC*	46	14.5	19	13.3	0.98	0.53–1.84	
Overdom	*GG-CC*	216	68.1	84	58.7	1.00		0.09
	*GC*	101	3.9	59	41.3	0.68	0.44–1.06	
Log-additive		–	–	–	–	0.83	0.62–1.12	0.23
Allele	*G*	443	70	189	66	1.00		0.20
	*C*	193	30	97	34	1.24	0.90–1.71	

*95%CI, 95% confidence interval; Codom, codominant; Dom, dominant; OR, odds ratio; Overdom, overdominant; Reces, recessive*.

### Italian cohort

Rate of genotyping success was 100% for all gene variants. The distribution of all genotypes was consistent with the HWE in both groups (*P* > 0.05), except for *THRH* rs7832552 in the control group (*P* = 0.04).

The results of genotype/allele frequency distributions as well as of binary logistic regression adjusted by sex are shown in Table 2 and summarized below. The allele (χ^2^ = 0.003, *P* = 0.95) and genotype frequency distributions of *THRH* rs7832552 did not differ between groups (χ^2^ = 0.26, *P* = 0.88) and no significant association was found between this polymorphism and EL using logistic regression adjusted by sex, or when analyzing both sexes separately (data not shown). Similar results were found for *IL6* rs1800795 (allele frequency: χ^2^ = 0.82, *P* = 0.36; genotype frequency: χ^2^ = 1.054, *P* = 0.59) and *ACSL1* rs6552828 (allele frequency: χ^2^ = 0.56, *P* = 0.46; genotype frequency: χ^2^ = 0.67, *P* = 0.72), with no significant association between these two polymorphisms and EL using logistic regression adjusted by sex, or when analyzing both sexes separately (data not shown).

**Table 2:** **Genotype/allele frequencies of *THRH* rs7832552, *ACSL1* rs6552828, and *IL6* rs1800795 and results of logistic regression analysis, in the Italian cohort**.

		**Controls**		**Cases**		**OR**	**95%CI**	***P*-value**
		**N**	**%**	**N**	**%**			
**rs7832552**								
Codom	*CC*	137	43.4	34	43.1	1.00		0.88
	*CT*	154	48.7	40	50.6	1.06	0.64–1.72	
	*TT*	25	7.9	5	6.3	0.81	0.26–2.28	
Dom	*CC*	137	43.4	34	43.0	1.00		0.96
	*CT-TT*	179	56.6	45	57.0	1.02	0.62–1.69	
Reces	*CC-CT*	291	92.1	74	93.7	1.00		0.64
	*TT*	25	7.9	5	6.3	0.77	0.30–2.14	
Overdom	*CC-TT*	162	51.3	39	49.4	1.00		0.76
	*CT*	154	48.7	40	50.6	1.09	0.68–1.75	
Log-additive	–	–	–	–	–	1.03	0.57–1.64	0.81
Allele	*C*	428	68	108	68	1.00		0.88
	*T*	204	32	50	32	0.98	0.67–1.43	
**rs6552828**								
Codom	*GG*	121	38.3	34	43.0	1.00		0.72
	*GA*	139	44.0	33	41.8	0.85	0.50–1.46	
	*AA*	56	17.7	12	15.2	0.77	0.35–1.55	
Dom	*GG*	121	38.3	34	43.0	1.00		0.44
	*GA-AA*	195	61.7	45	57.0	0.83	0.51–1.37	
Reces	*GG-GA*	260	82.3	67	84.8	1.00		0.59
	*AA*	56	17.7	12	15.2	0.85	0.43–1.65	
Overdom	*GG-AA*	177	56	46	58.2	1.00		0.72
	*GA*	139	44	33	41.8	0.92	0.54–1.51	
Log-additive	*–*	–	–	–	–	0.84	0.46–1.47	0.80
Allele	*G*	381	60	101	64	1.00		0.40
	*A*	251	40	57	36	0.86	0.56–1.23	
**rs1800795**								
Codom	*CC*	76	24.1	22	27.9	1.00		0.59
	*CG*	160	50.6	41	51.9	0.89	0.48–1.60	
	*GG*	80	25.3	16	20.2	0.70	0.34–1.45	
Dom	*CC*	76	24.1	22	27.8	1.00		0.48
	*CG-GG*	240	76.9	57	72.2	0.81	0.45–1.44	
Reces	*CC-CG*	236	74.7	63	79.8	1.00		0.35
	*GG*	80	25.3	16	20.2	0.75	0.41–1.39	
Overdom	*CC-GG*	156	49.4	38	48.1	1.00		0.84
	*CG*	160	50.6	41	51.9	1.06	0.64–1.75	
Log-additive	*–*	–	–	–	–	1.01	0.56–1.65	0.89
Allele	*C*	312	49	85	54	1.00		0.32
	*G*	320	51	73	46	0.84	0.57–1.19	

*95%CI, 95% confidence interval; Codom, codominant; Dom, dominant; OR, odds ratio; Overdom, overdominant; Reces, recessive*.

### Japanese cohort

Rate of genotyping success was as follows: *THRH* rs7832552, 97.0% in cases and 100% in controls; *IL6* rs1800795, 98.7% in cases and 100% in controls; *ACSL1* rs6552828, 95.9% in cases and 99.2% in controls. The distribution of all genotypes was consistent with the HWE in both groups (*P* > 0.05), except for rs6552828 in centenarians (*P* = 0.02).

The results of genotype/allele frequency distributions as well as of binary logistic regression adjusted by sex are shown in Table 3 and summarized below. We found no differences between cases and controls for *THRH* rs7832552 (allele frequency: χ^2^ = 0.012, *P* = 0.91; genotype frequency: χ^2^ = 0.592, *P* = 0.74), rs1800795 (allele frequency: χ^2^ = 0.07, *P* = 0.78; genotype frequency: χ^2^ = 0.07, *P* = 0.78), or rs6552828 (allele frequency: χ^2^ = 0.020, *P* = 0.89; genotype frequency: χ^2^ = 3.78, *P* = 0.15), with no significant association between any of the SNPs and EL using logistic regression adjusted by sex or when analyzing both sexes separately (data not shown).

**Table 3:** **Genotype/allele frequencies of *THRH* rs7832552, *ACSL1* rs6552828, and *IL6* rs1800795 and results of logistic regression analysis, in the Japanese cohort**.

		**Controls**		**Cases**		**OR**	**95%CI**	***P*-value**
		**N**	**%**	**N**	**%**			
**rs7832552**								
Codom	*CC*	135	27.1	191	26.3	1.00		0.87
	*CT*	230	46.1	351	48.3	1.06	0.81–1.41	
	*TT*	134	26.8	185	25.4	1.00	0.73–1.37	
Dom	*CC*	135	27.1	191	26.3	1.00		0.76
	*CT-TT*	364	72.9	536	73.7	1.04	0.80–1.35	
Reces	*CC-CT*	365	73.2	542	74.5	1.00		0.77
	*TT*	134	26.9	185	25.4	0.96	0.74–1.25	
Overdom	*CC-TT*	269	53.9	376	51.7	1.00		0.60
	*CT*	230	46.1	351	48.3	1.06	0.84–1.34	
Log-additive	*–*	–	–	–	–	1.00	0.85–1.17	0.99
Allele	*C*	500	50.1	733	50.4	1.00		0.99
	*T*	498	49.9	721	49.6	0.99	0.85–1.78	
**rs6552828**								
Codom	*AA*	186	37.6	247	34.6	1.00		0.12
	*GA*	229	46.3	369	51.8	1.19	0.92–1.53	
	*GG*	80	16.2	97	13.6	0.85	0.59–1.21	
Dom	*AA*	186	37.6	247	34.6	1.00		0.45
	*GA-GG*	309	62.4	466	65.4	1.10	0.86–1.40	
Reces	*AA-GA*	415	83.8	616	86.4	1.00		0.12
	*GG*	80	16.2	97	13.6	0.77	0.56–1.07	
Overdom	*AA-GG*	266	53.7	344	48.2	1.00		0.07
	*GA*	229	46.3	369	51.8	1.24	0.99–1.57	
Log-additive		–	–	–	–	0.98	0.82–1.16	0.77
Allele	*A*	601	61	863	61	1.00		0.78
	*G*	386	39	563	39	0.976	0.83–1.16	
**rs1800795**								
	*GG*	498	99.8	731	99.9	1		
	*GC*	1	0.2	1	0.1	0.84	0.05–14.09	0.9
Allele	*G*	997	100	1463	100			
	*C*	1	0	1	0			

*95%CI, 95% confidence interval; Codom, codominant; Dom, dominant; OR, odds ratio; Overdom, overdominant; Reces, recessive. Of note, logistic regression was not performed in the Japanese cohort for rs1800795 owing to the virtual absence of C-carriers in both groups*.

## Discussion

The main findings of our study are two-fold. First, all the studied SNPs showed functional significance, as reflected by the results of the luciferase constructs. This is the first attempt to determine (with an *in vitro* approach) the potential functional consequences of the rs16892496, rs7832552, and rs6552828 SNPs, with the *A*-allele, *T*-allele and *A*-allele up-regulating luciferase activity compared to the other alleles, respectively. The *THRH* rs7832552 and *ACSL1* rs6552828 SNPs are intronic genomic variants and, as such, could potentially alter the stability and/or alternative splicing of mRNA, as well as transcription factor binding (Tabor et al., 2002; Knight, 2005; Mercado et al., 2005; Sasabe et al., 2007). However, we found no association between *THRH* rs7832552, *IL6* rs1800795, and *ACSL1* rs6552828 and EL.

Although more research is obviously needed, we found no evidence that higher *THRH* expression (as theoretically associated with the *T*-allele or *CT-TT* genotypes vs. *CC*) might favor EL. Yet a GWAS study reported that the *TRHR* rs7832552 SNP was associated with lean body mass in US Caucasians (Liu et al., 2009). Subjects carrying the theoretically highest expressing (*TT*) genotype had 2.55 kg higher lean body mass compared to the other subjects. There is some scientific rationale in postulating that higher TRHR expression might help preservation of muscle mass in long-lived individuals: TRHR stimulates the hypothalamic-pituitary-thyroid axis, thereby leading to the release of thyroxin, a hormone that plays an important role in the development of skeletal muscle as well as in attenuating age-related changes in tissue function (Larsson et al., 1994). Although no association was found here, the *IL6* rs1800795 might be also a candidate to influence EL. Carriage of the “low-producing” *C*-allele has been positively associated with longevity in Turkish population (Kayaalti et al., 2011), whereas the high-producing *GG*-genotype has been linked with higher survival in elderly females from Sweden (Cederholm et al., 2007). On the other hand, the rs1800795 polymorphism has been linked with longevity in Italian centenarians from Treviso (Albani et al., 2009) although this finding was not replicated in other European cohorts (Di Bona et al., 2009) (including from other parts of Italy, Albani et al., 2009), or in US elders (Walston et al., 2009). As for *ACSL1* rs6552828, our data do not show an association of this polymorphism with EL despite the involvement of this gene in aerobic metabolism in the heart, liver, adipose and skeletal muscle tissues (Martin et al., 1997; Coleman et al., 2000; Hall et al., 2003; Mashek et al., 2006; Ellis et al., 2010). Recent research did not report an association between rs6552828 and an important age-related disease condition, the metabolic syndrome (Phillips et al., 2010).

A strength and novelty from our design stems from the use of a luciferase construct study to assess functionality of the 3 SNPs at the specific muscle tissue-level. However, our study has several limitations. First, besides the fact that we did not assess the functionality of the SNPs *in vivo* in blood samples and especially in muscle biopsies (which is understandable due to ethical reasons), we used convenience samples, which increases the risk of bias induced by population stratification. The SNPs rs1800795 and rs7832552 did not meet HWE in Spanish and Italian controls, respectively. In this regard, deviation from HWE does not necessarily reflect genotyping errors (Leal, 2005; Zou and Donner, 2006), with ~10% of all genotype–phenotype association studies actually showing failure of 1+ genotype distributions to meet HWE (Trikalinos et al., 2006). Second, we selected 3 SNPs based on previous GWAS, i.e., those showing associations of rs16892496 and rs7832552 with lean body mass (Liu et al., 2009) and of rs6552828 with VO_2peak_ (Bouchard et al., 2011). Besides differences in terms of population-specificity (ethic/geographic origin, age) between these 2 GWAS and our cohorts, an additional problem is that GWAS are generally successful to find very penetrant dominant genetic variants, but less useful to discover rarer variants, with a likely modest effect on some phenotypes, such as those that could be potentially associated with EL. Finally, a potential confounder of genetic association studies is differences in date of birth, e.g., the centenarians and controls of our study were born in the early 1900s and after 1930, respectively (Lewis and Brunner, 2004). In this regard, the potential demographic biases of longevity studies like ours performing cross-sectional comparisons of genotype/allele frequencies between controls and long lived individuals could be overcome by adding demographic information to genetic data (Yashin et al., 1999; Passarino et al., 2006; Dato et al., 2007). Thus, genetic–demographic methods should be applied in future studies in the field because they allow the estimation of hazard rates and survival functions in relation to candidate genes (Yashin et al., 1999; Passarino et al., 2006; Dato et al., 2007).

In summary, despite the potential functional consequences of the SNPs we studied (rs16892496, rs7832552, and rs6552828) none of them was associated with EL. Similarly, no association was found for rs1800795. More research is needed in the field with other cohorts, using larger population samples, as well as younger elderly (e.g., aged 65–85 years) to assess the potential link between these genetic variants and the human aging process.

### Conflict of interest statement

The authors declare that the research was conducted in the absence of any commercial or financial relationships that could be construed as a potential conflict of interest.
